# Squamous cell carcinoma arising in chronically damaged skin (Marjolin’s Ulcer): still an unmet need in the era of immunotherapy

**DOI:** 10.1093/oncolo/oyae326

**Published:** 2024-12-04

**Authors:** Mor Miodovnik, Yardenna Dolev, Roni Buchen, Miriam Rivka Brezis, Alla Nikolaevski-Berlin, Inbar Finkel, Ido Wolf, Inna Ospovat, Orit Gutfeld, Yasmin Leshem

**Affiliations:** Oncology Division, Tel Aviv Sourasky Medical Center, Tel Aviv, Israel; Oncology Division, Tel Aviv Sourasky Medical Center, Tel Aviv, Israel; Sackler Faculty of Medicine, Tel Aviv University, Tel Aviv, Israel; Oncology Division, Tel Aviv Sourasky Medical Center, Tel Aviv, Israel; Oncology Division, Tel Aviv Sourasky Medical Center, Tel Aviv, Israel; Oncology Division, Tel Aviv Sourasky Medical Center, Tel Aviv, Israel; Oncology Division, Tel Aviv Sourasky Medical Center, Tel Aviv, Israel; Sackler Faculty of Medicine, Tel Aviv University, Tel Aviv, Israel; Oncology Division, Tel Aviv Sourasky Medical Center, Tel Aviv, Israel; Oncology Division, Tel Aviv Sourasky Medical Center, Tel Aviv, Israel; Oncology Division, Tel Aviv Sourasky Medical Center, Tel Aviv, Israel; Department of Systems Immunology, Weizmann Institute of Science, Rehovot, Israel

**Keywords:** immunotherapy, PD-1, marjolin’s ulcer, cutaneous squamous cell carcinoma

## Abstract

**Background:**

Cutaneous squamous cell carcinoma (cSCC) is characterized by a high tumor mutational burden due to solar damage and a favorable response to anti-PD-1 immunotherapy. Yet, we encounter tumors arising in areas with minimal sun exposure, such as cSCC that develops in chronically inflamed skin, also known as Marjolin’s Ulcer (MU). The response of MU-SCC to immunotherapy remains unknown.

**Methods:**

We performed a retrospective analysis of patients diagnosed with cSCC and treated with cemiplimab or pembrolizumab in a single tertiary medical center. Patients lost to follow up were excluded.

**Results:**

Of the 84 eligible patients, 9 (11%) had MU-SCC. Of these, 2 (22%) reached partial response (PR), and none reached complete response (CR). In contrast, of the 75 patients with solar damage-related cSCC, 40 had PR (53%), and 20 had CR (26%). The difference between the two subtypes was significant (*P* < .001). Interestingly, 3 patients with MU-SCC received a second-line chemo-immunotherapy and experienced a partial response that continued for 5 to 21 months. Patients with MU-SCC had a significantly shorter median time to progression (TTP) (1.6 vs 51.6 months, *P* < .001) and progression-free survival (PFS) (1.6 vs 15.4 months, *P* < .001). Overall survival (OS) was not significantly shorter (17.4 vs 36.7 months, *P* = .096). Multivariate analysis confirmed that MU-SCC is an independent risk factor for shorter TTP (HR 5.5, 95% CI 2.2-14.0, *P* < .001) and PFS (HR 3.5, 95% CI 1.5-8.1, *P* = .003).

**Conclusions:**

This study suggests that immunotherapy is less beneficial in SCC-MU. More work is needed to verify our findings and explore other treatment options.

**Implications for practice**

This study highlights the differential response of squamous cell carcinoma originating from a Marjolin’s Ulcer (MU-SCC) to PD-1 blockade compared to other types of cutaneous SCC. This information can help set realistic expectations and facilitate informed decision-making regarding treatment options and outcomes. Patients with SCC-MU should be prioritized for participation in clinical trials.

## Introduction

Cutaneous squamous cell carcinoma (cSCC) is the second most common skin cancer, with a lifetime incidence estimated to be between 7% and 11%.^1,2^ The vast majority of cSCCs are cured by local excision or radiation. However, a small portion of the patients (3%-5%) will eventually develop a locally advanced or metastatic disease that requires a systemic approach.^3^ A few of the known risk factors are fair skin, old age, male gender, sun exposure, immune suppression, and chronically damaged skin.^4^

Marjolin’s ulcer (MU) is a term used to specify all cutaneous tumors that originate from chronically damaged skin including a burn scar, venous stasis, and chronic ulcers, such as in the setting of hidradenitis suppurativa (HS).^5,6^ MU is rare, but life-threatening due to its increased metastatic potential and high lethality. The pathogenesis of transformation from ulcer to malignancy is not fully understood but is believed to be related to chronic signaling of inflammation.^7^ The most common histology of MU is squamous cell carcinoma (MU-SCC).

Treatment with programmed cell death-1 (PD-1) blockade has become the preferred first-line treatment for unresectable or metastatic cSCC. This recommendation is based on a few single-armed clinical trials showing a high response rate that outperforms the historical response rate to chemotherapy with a favorable profile of side effects.^8,9^ It is believed that the high mutational burden caused by exposure to ultraviolet radiation leads to the recognition of neoantigen by immune cells and primes the effect of PD-1 blockade. It is currently unknown if cSCC that arises due to other mechanisms of damage can recapitulate these impressive responses. The use of PD-1 blockade in MU-SCC was reported in a case series.^10^

The purpose of this study is to present real-world data from a large tertiary clinical center, comparing the response rate to PD-1 blockade in MU-SCC with that observed in other cSCC subtypes.

## Methods

### Setting and patients

This is a retrospective, single-center, observational study conducted at Tel Aviv Sourasky Medical Center, a tertiary cancer center in Israel. Consecutive records of all adult patients (age > 18) diagnosed with cSCC who were prescribed cemiplimab or pembrolizumab were identified in the electronic database and reviewed. Patients who did not receive the medications or failed to return to their oncologist for a post-treatment clinical evaluation were excluded from the study. The study was approved by the local Helsinki regulatory ethics committee (identifier: 0618-19 TLV).

## Data review

Medical records of patients with cSCC starting cemiplimab or pembrolizumab between February 2018 and November 2023 were reviewed. Patients’ outcomes were last updated in July 2024. We collected data on the demographic and clinical characteristics including age, gender, Eastern Cooperative Oncology Group (ECOG) performance status (PS), date of diagnosis, location of primary and metastatic disease, and predisposition of non-healing chronic ulcer (MU). Patients who were documented with past excision of cutaneous SCC at a different location or had multiple concurrent cutaneous SCC were considered to have recurrent SCC. In accordance with ESMO guidelines for real-world data reporting in oncology^11^ response to therapy was based on the clinician’s qualitative description as it was documented in the electronic medical records. Real-world time to progression (TTP) was defined as the time from the first day of treatment to the day of a documented disease progression. Real-world progression-free survival (PFS) was defined as the time from the first day of treatment to the day of documented progression or death. Overall survival (OS) was defined as the time from the first day of treatment to the day of documented death from any cause and extracted from the population registry bureau.

## Statistical analysis

We applied descriptive statistics including mean and its 95% CI for continuous variables and frequencies for categorical variables. To assess if a difference between groups was significant, we applied the Mann-Whitney test for continuous variables and the 2-sided Fisher’s exact test for categorical variables. OS and PFS were evaluated by the Kaplan-Meier method. The log-rank test was used to determine if the differences between two groups were statistically significant. The Cox proportional-hazards regression model was performed to determine the influence of multiple variables including MU-SCC on PFS and OS. The GraphPad Prism and SAS software were used in this study.

## Results

### Demographic and clinical characteristics of our cohort

Of 110 patients with cSCC prescribed cemiplimab or pembrolizumab at our medical center, 97 received the treatment, and 84 were eligible for inclusion in our study. Of 13 patients excluded due to loss to follow-up, 11 were recorded by the population registry bureau as deceased, with a median OS of 3.5 months (SD 3.7 months).

As shown in Table 1, the mean age in the entire cohort was 79 years (95% CI 77-82), and it was male predominant (70%). The majority of our cohort (71%) had a good performance status of zero or one. The average time from cSCC diagnosis to immunotherapy initiation was 22 months (95% CI 14-30). Thirteen patients (16%) had concurrent hematological malignancy, and one patient had a concurrent diagnosis of human immunodeficiency virus (HIV). Forty patients in our cohort (52%) had lymph node involvement, and 10 (14%) had more distant metastasis including 10 (12%) with lung metastases, 3 (4%) with bone metastases, and one (1%) with brain metastases.

**Table 1.** Characteristics of patients.

	MU-type *N* = 9	Non-MU-type *N* = 75	P	All *N* = 84
Age (mean, 95% CI)	64 (49-79)	81 (79-83)	.003	79 (77-82)
Male gender, *N* (%)	4 (44%)	55 (73%)	.118	59 (70)
Time from diagnosis, mean in months (95% CI)	5 (2-9)	24 (15-33)	.07	22 (14-30)
Below the umbilicus, *N* (%)	6 (67)	8 (11)	.001	14 (17)
Past other cSCC vs first, *N* (%)	1 (11)	20 (27)	.435	21 (26)
Hematological malignancies, *N* (%)	0 (0)	13 (17%)	.343	13 (16)
First-line *N* (%)	8 (89)	65 (87)	1.000	73 (87)
Past radiation, *N* (%)	1 (13)	21 (28)	.676	22 (27)
Cemiplimab, *N* (%)	8 (89)	70 (93)	.505	78 (93)
ECOG PS 0-1, *N* (%)	4 (50)	52 (74)	.212	55 (71)
Locally advanced (LND+), *N* (%)	4 (50)	36 (52)	1.00	40 (52)
Distant met (non LND), *N* (%)	1 (14)	9 (13)	1.00	10 (14)

Abbreviations: MU, Marjolin’s ulcer; cSCC, cutaneous squamous cell carcinoma; ECOG PS, Eastern Cooperative Oncology Group performance status; LND +, lymph node positive.

## Demographic and clinical characteristics of patients with MU-SCC

Of 84 eligible patients, 9 (11%) had cSCC that developed in MU. Patients with MU-SCC were younger (64 vs 81 years, *P* = .003). Four patients from the MU-SCC subtype were male (44%), compared to 55 (73%) in the non-ulcer type (*P* = .118). The average time from diagnosis to treatment was 5 months in the MU-SCC type, compared to 24 months in the non-ulcer type (*P* = .07). The majority of MU-SCC (67%) were located at the lower part of the body, below the umbilicus, while in the non-ulcer type most were located in sun-exposed areas (89%) above the umbilicus (*P* = .001). No significant differences were found in the rate of good performance status of zero or one, concurrent hematological malignancy, a history of other cSCC, a history of local radiation, rate of lymph node involvement, or the rate of distance metastases (Table 1). Three patients ultimately underwent surgery either due to disease progression (*n* = 2) or as part of a neoadjuvant regimen (*n* = 1). Among them, one patient, with a MU-SCC subtype located on the foot had the tumor successfully removed with no recurrence observed during 14 months of post-surgical follow-up.

## Clinical outcomes of patients with MU-SCC

**Figure 1.**
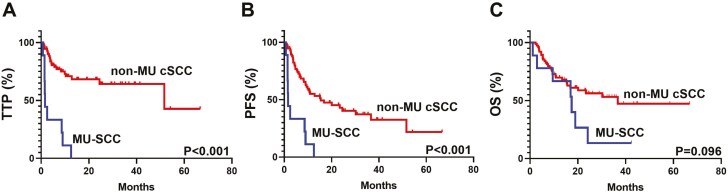
Kaplan-Meier curves for (A) time to progression (TTP), (B) progression-free survival (PFS), and (C) overall survival (OS) of patients with MU-SCC and non-MU cSCC receiving pembrolizumab or cemiplimab.

The median time to follow-up at the data cutoff was 18 months. Of the 9 patients with MU-SCC, only 2 (22%) had a partial response, and none achieved a complete response (Table 2). The response rate in the non-MU type cSCC was 80%, with 40 patients (53%) reaching partial response and 20 (27%) complete response, including one patient with HIV. The difference in the response pattern was statistically significant (*P* < .001). As shown in Figure 1A, the median TTP was 1.6 months for the MU-SCC subtype and 51.6 months for the non-MU cSCC (*P* < .001). The median PFS was 1.6 months for the MU-SCC subtype and 15.4 months for the non-MU cSCC (*P* < .001). Overall survival was not significantly different between the two subtypes (17.4 vs 36.7 months, *P* = .096). Seven out of 9 (78%) patients with MU-SCC died during follow-up, compared to 31 out of 75 (41%) patients in the non-MU cSCC cohort (*P* = .072). Notably, in the MU-SCC subtype, all patients who died experienced disease progression. In contrast, in the non-MU cSCC subtype, only 10 out of 31 patients (43%) who died had documented disease progression. This suggests that in this older subtype of cSCC, progression is not the predominant cause of death. Following progression, 3 patients with MU-SCC received a second-line combination of carboplatin or cisplatin with cemiplimab, and all 3 had a partial response lasting between 5 and 21 months. Out of 5 patients with non-ulcer type cSCC that received second-line therapy, 3 received a combination of carboplatin or cisplatin with cemiplimab and all reached a partial response lasting for 3 to 8 months. Another treatment modality that was used upon progression was radiotherapy. Of the patients with MU-SCC, 2 received radiotherapy upon progression. One showed no response, and another a short-term partial response lasting for 4 months. In the non-MU cSCC, 6 patients received radiotherapy, of which one achieved stable disease, 4 partial responses, and one complete response.

**Table 2.** Response pattern.

	MU-type *N* (%)	Non-MU-type *N* (%)	*P*
No response	7 (78)	15 (20)	<.001
Partial response	2 (22)	40 (53)	
Complete response	0 (0)	20 (27)	

## Association between MU-SCC and outcome

A multivariate analysis was conducted using Cox proportional hazards regression model to investigate the correlation between the MU-SCC subtype and the patient’s outcome while excluding the possibility of confounding variables mediating this effect (Tables 3 and 4). The variable distant metastases was not included in the multivariate analysis for TTP because of lack of convergence of the model, and the variable type of PD-1 blockade was not included in the multivariate analysis for PFS and OS because of lack of convergence of the model. We found that the predisposition of a non-healing ulcer was a strong and independent predictor of shorter TTP (HR 5.5, 95% CI 2.2-14.0, *P* < .001) and PFS (HR 3.5, 95% CI 1.5-8.1). In other words, patients with MU-SCC faced a 5.5-fold higher risk of progression, and their combined risk of progression or death was 4 times greater compared to that of patients with non-MU cSCC. The primary location of the SCC only marginally correlated with the median PFS (*P* = .049). All other explanatory variables did not significantly affect the TTP or PFS. We found that a good PS of zero or one was the only independent predictor of prolonged OS (HR 0.33, 95% CI 0.16-0.67, *P* = .002). The influence of all other variables on OS, including the predisposition of a non-healing ulcer (*P* = .589) was not significant.

**Table 3.** Multivariate analysis for time to progression.

	TTP	
	HR (CI 95%)	*P*
MU-SCC vs other cSCC	5.51 (2.18-13.96)	<.001
Male vs female	2.04 (0.63-6.65)	.238
Age ≤ 80, vs > 80	0.78 (0.29-2.16)	.638
Cemiplimab vs pembrolizumab	1.12 (0.12-10.72)	.921
Past radiation vs none	1.32 (0.41-4.26)	.645
Below vs above the umbilicus	1.66 (0.66-4.19)	.283
Past other cSCC vs first	0.34 (0.07-1.58)	.168
Concurrent hematological malignancy vs none	0.96 (0.23-3.96)	.951
Locally advance (LND+) vs none	0.84 (0.29-2.42)	.749
ECOG PS 0-1 vs 2-4	0.48 (0.16-1.44)	.189

Abbreviations: cSCC, cutaneous squamous cell carcinoma; ECOG PS, Eastern Cooperative Oncology Group performance status; HR, hazard ratio; LND+, lymph-node positive; MU, Marjolin’s ulcer.

**Table 4.** Multivariate analysis for progression-free survival and overall survival.

	PFS		OS	
	HR (CI 95%)	*P*	HR (CI 95%)	*P*
MU-SCC vs other cSCC	3.53 (1.53-8.10)	.003	1.28 (0.51-3.23)	.598
Male vs female	1.41 (0.58-3.45)	.451	1.50 (050-4.57)	.470
Age ≤ 80, vs > 80	0.85 (0.39-1.87)	.682	0.59(0.24-1.49)	.266
Past radiation vs none	1.01 (0.44-2.31)	.993	0.74 (0.27-2.04)	.555
Below vs above the umbilicus	3.81 (1.01-14.40)	.049	1.24 (0.24-6.59)	.798
Past other cSCC vs first	0.60 (0.24-1.49)	.273	0.63 (0.21-1.89)	.412
Concurrent hematological malignancy vs none	0.70 (0.24-2.03)	.510	1.18 (0.38-3.67)	.771
Locally advance (LND+) vs none	0.70 (0,33-1.50)	.362	0.72 (0.28-1.85)	.490
Distance metastases vs none	0.27 (0.06-1.27)	.097	0.59 (0.11-3.06)	.530
ECOG PS 0-1 vs 2-4	0.58 (0.31-1.11)	.101	0.33 (0.16-0.68)	.002

Abbreviations: MU, Marjolin’s ulcer; HR, hazard ratio; cSCC, cutaneous squamous cell carcinoma; LND+, lymph-node positive; ECOG PS, Eastern Cooperative Oncology Group performance status.

## Discussion

In this study, we found that MU-SCC was a strong and independent predictor of shorter TTP and PFS, while all other variables had no significant effect of their own. To the best of our knowledge, this is the first retrospective study to systematically compare the response rate (RR) of MU-SCC to other types of cSCC.

The pathophysiology of MU has been discussed for over 100 years. Various etiological factors are responsible for malignant transformation. These include areas of chronic scar tissue that are less populated by immune cells, resulting in avoidance of immune detection.^12,13^

While 89% (67 of 75) of the non-ulcer type cSCC in our cohort were located in sun-exposed areas, most SCC-MU were located below the umbilicus (6 of 9, 67%), where solar damage is less frequent. cSCC are characterized by a high tumor mutation burden (TMB) due to a cumulative exposure to UV radiation leading to DNA damage.^4^ A possible explanation for the low response rate of MU is a lower mutational load resulting from non-UV-related pathogenesis. Shalhout et al^10^ described a case series of 5 patients with MU-SCC who received PD-1 blockade. Similar to our cohort, the tumor location of all 5 patients was below the umbilicus. While this study noted a superior RR compared to our findings, with 3 out of 5 patients achieving a partial response, only one persisted for more than 10 months, indicating a generally unfavorable prognosis.

Locally advanced or metastatic cSCC posed a significant therapeutic challenge until the advent of immunotherapy. In the pre-immunotherapy era, the duration of response to chemotherapy and epidermal growth factor receptor (EGFR) inhibitors was only several months. A few phase II clinical trials introduced PD-1 blockade, showing a remarkable disease control rates, thereby changing the treatment guidelines.^14,15^

The RR of cemiplimab in the EMPOWER-CSCC1 study ranged from 41% to 49%,^16^ while the RR to pembrolizumab in the KEYNOTE-629 study was 51%.^17^ A higher RR of 68% was found in a phase II clinical trial that evaluated neoadjuvant cemiplimab in locally advanced or resectable stage IV cSCC.^18^ In contrast to these previous data, the RR in our cohort was 70% in the entire cohort and 80% when excluding the patients with MU-SCC. The high response rate may be attributed to patient selection for immunotherapy at our medical center (with only 12% having distant metastasis) or may be due to our reliance on clinical assessment that may overestimate the treatment benefit.^19^ The high response rate in our cohort aligns with a real-world study performed in Israel showing a response rate of 80% among patients with cSCC treated with PD-1 blockade.^20^

We explored the combination of immunotherapy with chemotherapy (ie, chemo-immunotherapy) in MU-SCC not responding to PD-1 blockade as a single agent in a few of our patients. Chemotherapies can augment tumor immunity, mostly by inducing immunogenic cell death, debulking the tumor mass, and depleting inhibitory immune cell populations.^21^ Based on the poor RR to monotherapy PD-1 blockade in our cohort, we suggest further investigation of chemo-immunotherapy in the setting of MU-SCC. Another treatment option that needs to be further explored is the combination of radiotherapy and PD-1 blockade. However, in our cohort, the 2 patients who received radiation following disease progression experienced minimal benefit.

Our findings of young age at disease onset (81 years vs 64 years) in the MU cohort align with previous reports.^5^ This suggests that the latency of cancer formation for MU is shorter than that of solar injury, which might indicate a more aggressive tumor biology. Actinic damage-related SCC is driven by the accumulation of DNA mutations over years of sun exposure while the pathogenesis of tumors arising on a background of chronic inflammation is different. Interestingly, only a trend toward shorter OS in the MU-SCC was demonstrated. As non-MU patients were older, their survival may be more dependent on age-related morbidity. Indeed, 21 of 31 patients with non-MU who died, did not have a disease progression. All 7 patients with MU who died were documented with disease progression. Because of the retrospective nature of our study, the cause of death was not consistently documented, thus cancer-specific survival was not calculated. Our cohort was not powered to clarify if the difference in all-cause mortality between the two subtypes is significant.

Our trial has a few limitations. It is a single-center study with a relatively small sample size. Thus, further generalization of our findings will require validation. The retrospective nature of the study can amplify the influence of unknown confounders and introduce bias inherent to this study design. Patients excluded due to loss to follow-up could have skewed our cohort toward a higher proportion of responders.

Altogether, this study is the first to compare the response rate of MU-SCC to other types of cSCC and shows a very poor RR in this subtype. If further validated in larger cohorts, exploring alternative treatment strategies is warranted to enhance efficacy.

## Data Availability

The data underlying this article will be shared on reasonable request to the corresponding author.
